# Utilization of Noxious Weed Water Hyacinth Biomass as a Potential Feedstock for Biopolymers Production: A Novel Approach

**DOI:** 10.3390/polym12081704

**Published:** 2020-07-29

**Authors:** Rijuta Ganesh Saratale, Si-Kyung Cho, Gajanan S. Ghodake, Han-Seung Shin, Ganesh Dattatraya Saratale, Yooheon Park, Hee-Seok Lee, Ram Naresh Bharagava, Dong-Su Kim

**Affiliations:** 1 Research Institute of Biotechnology and Medical Converged Science, Dongguk University-Seoul, Ilsandong-gu, Goyang-si, Gyeonggido 10326, Korea; rijutaganesh@gmail.com; 2 Department of Biological and Environmental Science, Dongguk University, Ilsandong-gu, Goyang-si, Gyonggido 10326, Korea; sk.cho@dongguk.edu (S.-K.C.); ghodakegs@gmail.com (G.S.G.); 3 Department of Food Science and Biotechnology, Dongguk University-Seoul, Ilsandong-gu, Goyang-si, Gyeonggido 10326, Korea; spartan@dongguk.edu (H.-S.S.); ypark@dongguk.edu (Y.P.); 4 Department of Food Science and Technology, Chung-Ang University, Anseong 17546, Korea; hslee0515@cau.ac.kr; 5 Department of Microbiology, School for Environmental Sciences Babasaheb Bhimrao Ambedkar University, Vidya Vihar, Uttar Pradesh 226025, India; bharagavarnbbau11@gmail.com; 6 Department of Environmental Science and Engineering, Ewha Womans University, Seoul 120-750, Korea; dongsu@ewha.ac.kr

**Keywords:** water hyacinth biomass, peracetic acid pretreatment, alkaline pretreatment, poly(3-hydroxybutyrate), *Ralstonia eutropha*, enzymatic hydrolysis

## Abstract

This study aims to utilize a noxious weed water hyacinth biomass (WH) for polyhydroxybutyrate (PHB) production. Alkaline and peracetic acid pretreatment was employed for the hydrolysis of WH and consequently enzymatic saccharification to produce fermentable sugars for PHB production. The pretreatment competence was determined using various operational parameters. By applying ambient conditions, alkaline pretreatment gave higher lignin removal of 65.0%, with 80.8% hydrolysis yield, and on enzyme hydrolysis (40 FPU/g of dry WH), produced total reducing sugar of about 523 mg/g of WH. The resulted WH enzymatic hydolysates were evaluated for the production of PHB by *Ralstonia eutropha* (ATCC 17699). The WH hydrolysates cultivation was compared to synthetic hydrolysates that contain a similar carbon composition in terms of bacterial growth and PHB synthesis. The effects of various supplements to enhance PHB production were estimated. Supplementation of corn steep liquor (CSL) as a cheap nitrogen source with WH hydrolysates favored a higher amount of PHB synthesis (73%), PHB titer of 7.30 g/L and PHB yield of 0.429 g/g of reducing sugar. Finally, using standard analytical tools, the physical and thermal characteristics of the extracted PHB were evaluated. The findings revealed WH was a promising and technically feasible option for transforming biomass into sustainable biopolymer conversion on a large scale.

## 1. Introduction

Durability and extraordinary mechanical and thermal properties make conventional plastics useful for many applications. However, they are not readily biodegradable and thus accumulate in the environment and directly lead to waste disposal problems [1]. There is a well-built indication that synthetic polymer production (300 Mt/year) and ordinary management practices have caused irretrievable harm to the environment [2]. Considering the rising demand for energy and decline of fossil resources, the global economy is currently aiming to replace recognized energy sources by greener, bio-based, and sustainably produced equivalents [3,4]. Biopolymers, notably polyhydroxyalkanoates (PHAs), can address numerous global environmental problems, for instance, ocean pollution and greenhouse gas emissions, as they can biodegrade into harmless products in various environments such as home compost, anaerobic digestion, and marine environments within a short time. Biodegradability makes PHAs an ideal substitute for conventional polymers in single-use products [5,6]. Moreover, PHA polymers can be easily improved both by modifying functional groups and by creating physical mixtures which increase their functionality for different applications. Thus anticipated, a significant development can be foreseen in the short- and long-term future for bio-based products [7].

Polyhydroxyalkanoates (PHAs) are polyesters that are produced during the secondary metabolism of bacteria and archaea in the presence of excess carbon sources and insufficiency of nutrients [1,6]. In the PHAs group, polyhydroxybutyrate (PHB) is gaining enormous consideration due to extraordinary characteristics, such as a higher degree of crystallinity, tensile strength, and melting temperature (175 °C) with desirable water, and gas barrier properties [8]. It was supposed that bioplastics will contribute 25 to 30% of the total plastics market worldwide [2]. However, one major challenge for industrial production is the reliable and scalable supply of carbon sources, as it makes up to 48% of PHB production costs [2,9]. In sustainable PHB production using non-edible, abundant and renewable carbon sources such as waste biomass, agricultural residues will not only ensure agreement with zero waste policies but also enable upcycling of the generated wastes within biorefineries to high value products [4,6,10]. PHBs at minimum cost have maximum societal benefits and far lower environmental impacts than their petrochemical counterparts [11].

*Eichhornia crassipes*, commonly known as water hyacinth (WH), is a rapid growing perennial aquatic noxious weed widely distributed in tropical and subtropical areas worldwide. Because of a higher growth rate (220 kg/ha/day) it forms a large mat on the surface of water and severely deteriorates the aquatic ecosystem [12,13]. However, WH biomass is primarily composed of high holocellulose (44–66.9% of dry matter) with a lower amount of lignin [14]. Thus, WH can be considered a promising and sustainable biomass resource that does not compete with food and has many advantages from environmental and economic perspectives for biopolymer production [9,10]. However, WH biomass requires pretreatment to break down the structure and delignification of biomass. Lignin acts as a major obstacle for enzymatic accessibility and thus delignification acts as a key control step to achieve significant saccharification of WH biomass [15]. Furthermore, selection and effectiveness of the pretreatment method also depends on the type of biomass [16]. Alkaline pretreatment is well-known for the effective delignification of biomass by cleaving the bonds between hemicelluloses and lignin, thereby increasing the porosity of biomass and facilitating the accessibility of biomass to hydrolytic enzymes [17]. Moreover, peracetic acid (PA) acts as a potent oxidizing agent with the ability to destruct the lignin structure by cleaving side chains and β aryl bonds and oxidizing the aromatics in lignin. The depolymerized lignin fragments are soluble in water and make holocelluose easily accessible for enzymatic hydrolysis [18,19].

The objectives of the present study focus on exploring the chemical pretreatments of WH biomass and to determine the suitable conditions favoring delignification and better saccharification after enzymatic hydrolysis. The physicochemical modifications in WH after pretreatment were studied using different analytical tools. Further, the resulting WH biomass hydrolysates and their feasible conversion for biopolymer production by *R. eutropha* were investigated. The effects of nutrient supplements to maximize PHB production were assesed. Chemical structure and crystallinity of the extracted biopolymer was determined by use of analytical techniques such as Fourier transform infrared spectroscopic (FTIR) and X-ray diffraction spectroscopic (XRD), respectively. Thermal properties of the biopolymer were examined by thermo gravimetric analysis (TGA) and differential scanning calorimetry (DSC) analysis. The focus is on finding the best experimental production methods for sustainable biopolymer production using WH biomass with regard to recommending it for future industrial applications.

## 2. Materials and Methods

### 2.1. Water Hyacinth Biomass and Chemicals

Fresh water hyacinth plants were collected from the aquatic habitats of Panchganga River, Kolhapur, India. The whole plant bodies without roots were used for the studies. Firstly, the plants were washed with tap water to get rid of dirt, sliced into small pieces and further dried in an oven by maintaining the temperature at 70 °C until constant weight was achieved. The dried biomass was milled and sieved to get 0.3–0.4 mm particle size, and stored in air-tight containers at 4 °C. Sodium hydroxide (NaOH), acetic anhydride, 30% H_2_O_2_, corn steep liquor (CSL), Whatman filter paper No. 1, and *Trichoderma reesei* cellulase (Celluclast 1.5 L) were procured from Sigma–Aldrich (St. Louis, MO, USA). Supplements such as cotton seed cake and ground nut cake were purchased from a local animal feed supplier in India. Other required chemicals used for pretreatment, PHB production, and for other experiments, including characterization studies, were of Analytical grade.

### 2.2. Pretreatment Studies and Enzymatic Hydrolysis

To hydrolyze and to achieve better saccharification after enzymatic hydrolysis, WH biomass was pretreated with NaOH and peracetic acid. Biomass exposed to auto-hydrolysis at neutral pH exclusive of any chemicals acted as a control. Initially, WH biomass was subjected to NaOH (2%) solution and heated in an electrical water bath at 100 °C for 3 h. Similarly, WH biomass was added in freshly prepared peracetic acid solution (a mixture of equivalent concentration of acetic anhydride and 30% H_2_O_2_) and further heated at 100 °C in an electric water bath for 3 h. The effects of chemical (NaOH) dosage (*w*/*v*) (1%, 2%, 3%, and 4%), WH biomass solid to liquid ratio concentration (*w*/*v*) (1:25, 1:20, 1:10, and 1:8), pretreatment temperature (RT, 60 °C, 80 °C and 100 °C) and pretreatment time (2 h, 3 h, 4 h, and 6 h), were steadily investigated. During the optimization study, the “one variable factor at a time” methodology was used. For each pretreatment, the proportion of the WH biomass in reaction mixture kept at 1:20. After each pretreatment, the WH biomass was separated using a vacuum and rinsed with distilled water until the solution became a neutral pH. The obtained WH biomass further dried at 60 °C until constant weight and finally preserved in plastic bags at 4 °C for further biomass chemical composition and structural analysis. Enzymatic hydrolysis of untreated and pretreated WH biomass was performed following the protocol reported previously [8]. To achieve better saccharification, the optimum substrate (WH; 5 to 20 g/L) and enzyme concentration (20–50 FPU/g of WH) were determined.

### 2.3. PHB Production Using WH Hydrolysates by R. eutropha

The PHB producing strain *Ralstonia eutropha* (ATCC 17699) was procured from ATCC (Manassas, VA, USA). The bacterial strain was grown in shake flasks consisting of Tryptic Soy Broth lacking dextrose (TSB; Becton Dickinson, Heidelberg, Germany) at 200 rpm. PHB biosynthesis in batch fermentation was carried out by inoculating 5% (*v*/*v*) seed culture in the minerals salt medium (Table 1) to an initial volume of 200 mL with WH hydrolysates. The pH of production medium was maintained at 7.0 and kept at 30 °C for 36 h under shaking condition. The synthetic hydrolysate (SH) with a similar concentration of carbon composition (mainly glucose, xylose and arabinose) was prepared as in WH hydrolysate and further studied the bacterial growth and PHB production performance. For the SH, glucose, xylose and arabinose were steadily dissolved in distilled water by heating the mixture in a microwave for 20 s until the solubility improved. The bacterial growth and PHB synthesis kinetic parameters were deliberated with the procedure described previously [17]. All parameters were conducted in three separate sets, and median values of PHB synthesis parameters were deliberated.

#### Effects of Supplementation of Cheap N Sources on PHB Production

The effects of supplementing different cheap nitrogen sources, mainly corn steep liquor (CSL), cotton seed cake (CSC) and ground nut cake (GNC), using alkaline pretreated WH enzymatic hydrolysates (20 g/L) on bacterial cell proliferation and PHB production kinetic studies were consistently investigated.

### 2.4. Analytical Methods

Cellulose, hemicellulose and lignin concentrations of untreated and pretreated WH biomass were determined [20]. On enzymatic hydrolysis of WH biomass, the obtained reducing sugar (RS) concentration was checked by the dinitrosalicylic acid method [21]. Hydrolysis and glucose yield was determined by using the formulas described earlier [15]. The existence of fermentable sugars (mainly glucose, xylose, and arabinose) in WH hydrolysates was estimated using high performance liquid chromatography (HPLC).

The structural transformation of raw and pretreated WH biomass was studied using a SEM JEOL JSM-6360A microscope (Tokyo, Japan) and applying a beam increasing voltage of 20 kV. The FT-IR analysis accomplished from 4000 cm^−1^ to 400 cm^−1^ with a usual scan of 16 scans at a resolution of 4 cm^−1^, using Fourier-transform infrared spectroscopy (Cary 630; Agilent, Santa Clara, CA, USA). X-ray diffraction (XRD) analysis of WH biomass was conducted by a D2 Phaser tabletop model at 30 kV (Bruker, Billerica, MA, USA). The crystallinity index (CrI) was assessed from the obtained XRD details and calculated by employing the definitive peak height technique [22].

### 2.5. PHB Extraction and Analytical Characterization

The dry cell weight concentration (DCW; g/L) was determined gravimetrically after centrifugation for 10 min at 4 °C. The supernatant was removed and the separated cell biomass was washed and freeze-dried to constant weight. The PHB content (% g/g) was calculated by PHB (g/L) per DCW (g/L). Total sugar concentration was calculated as the sum of glucose (g/L), xylose (g/L) and arabinose (g/L). The substrate consumption (glucose, xylose, and arabinose) was calculated as the difference between the substrates added and substrate measured adjusted for the change in volume. PHB extraction from the lyophilized cell powder was conducted through dispersion of chloroform in a sodium hypochlorite solution followed by precipitation using 80% methanol to separate PHB by filtration [17]. To attain the purified PHB, the obtained pellet was repeatedly dissolved two times in methanol-chloroform mixture, air-dried and measured gravimetrically. The physicochemical characteristics of the extracted PHB were analyzed by XRD and FTIR, keeping the same condition mentioned in Section 2.4.

### 2.6. Thermal Analysis and Molecular Mass Determination of Produced PHB

The purified biopolymer was dried out into a fine powdered form to carry out the thermal analysis. TGA was carried out under a nitrogen atmosphere at the heating rate of 20 °C/min to determine the thermal stability, degradation pattern and melting point of the synthesized polymer (Hi-Res TGA 2950, TA Instruments, New Castle, DE, USA) [23]. The melting temperature (*T_m_*) and glass transition temperature (*T_g_*) were measured by differential scanning calorimetry (DSC, model 2920, TA Instruments, New Castle, DE, USA). The temperature aligns for DSC varied as of 50 °C to 250 °C and the heating and cooling rate was retained at 10 °C/min and 5 °C/min, respectively.

### 2.7. Statistical Analysis

The obtained results were determined using one-way analysis of variance (ANOVA) followed by using Tukey’s HSD test in the software of Graph Pad In-Stat version 3.06 (GraphPad Software Inc., CA, USA). A threshold of *p* = 0.05 was considered significant to evaluate differences between means.

## 3. Results and Discussion

### 3.1. Effects of Chemical Pretreatment on Enzymatic Hydrolysis of WH

Water hyacinth is regarded as a malicious aquatic weed because of its speedy proliferation and ecological adaptability which creates severe impacts on the aquatic ecosystem and socio-economic progress [12,24]. Utilization of WH biomass as a raw material for biopolymer production is an attractive solution to mitigate the environmental pollution and waste disposal management at higher levels. In WH biomass, mainly cellulose and hemicellulose components can be converted into fermentable sugars after enzymatic hydrolysis. However, lignin acts as a barrier and hinders the enzymatic digestibility of biomass. To overcome this, it is significant to pertain an appropriate pretreatment. In this study, initially WH biomass was exposed to NaOH and peracetic acid (2% each concentration) at 100 °C for three hours and their effect was compared in terms of WH biomass constituent composition, delignification and enzymatic digestibility. NaOH pretreatment showed higher delignifcation (50.2%) and gave significant hydrolysis yield (64.3%), glucose yield (75.0%) and saccharification yield (418 mg/g of WH) after enzymatic hydrolysis, which is significantly higher than peracetic acid pretreatment.

Table 2 represents the comprehensive results of chemical composition, lignin removal and enzymatic digestibility of WH before and after each pretreatment. This is in line with previous research results, in which it was stated that the alkaline pretreatment under certain conditions exhibited higher delignification, making holocellulose more accessible to hydrolysis and leading to significant saccharification yield [25].

The enhancement in cellulose concentration after NaOH pretreatment might be due to the delignification and solubilization of hemicellulose components [26]. The foregoing results suggest that alkaline pretreatment exhibited higher delignification and better saccharification yield, so a further study was performed with NaOH pretreatment.

### 3.2. Determination of the Best Pretreatment Conditions

**Figure 1:**
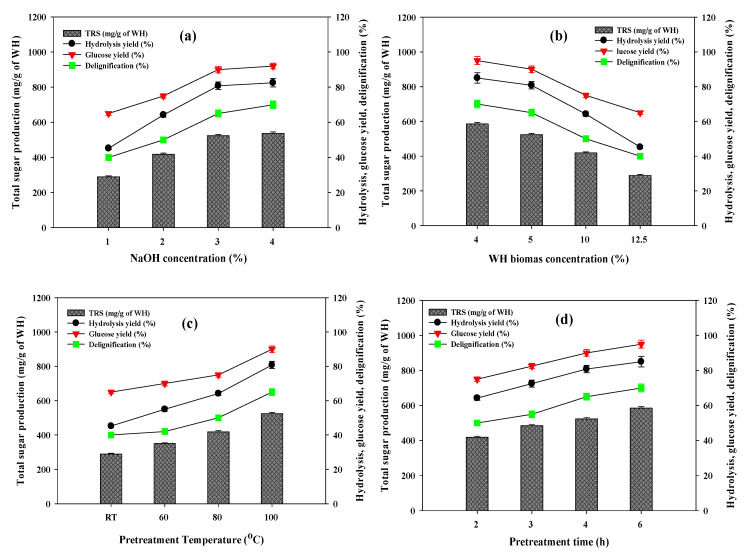
Effects of (**a**) different NaOH concentration, (**b**) WH biomass loading, (**c**) pretreatment temperature and (**d**) pretreatment time on delignification, total reducing sugar, hydrolysis yield, and glucose yield from WH after enzymatic hydrolysis.

To enhance the saccharification of WH, further studies were devoted towards the optimization of pretreatment conditions, mainly NaOH concentration, WH biomass loading, pretreatment temperature and pretreatment incubation time. The maximum delignification (65%) and significant hydrolysis yield (80.8%) of WH biomass was recorded at 3% NaOH pretreatment with 5% substrate loading at 100 °C for 4 h of incubation (Figure 1). During the optimization of chemical loading at 3% and 4% NaOH, there was a slight increase in delignification; however, no significant improvement in the saccharification of WH biomass was observed (Figure 1a).

Whereas, up to 5% WH biomass loading exhibited significant lignin removal and saccharification yield but further increases lead to sharp declines in both performances (Figure 1b). This might be due to the chemical dosage (3%) being insufficient to hydrolyze and delignify the WH biomass effectively at higher concentrations (>5%). Considering this, 5% WH biomass and 3% chemical dosage were selected for further studies. Pretreatment temperature of 100 °C was found to be optimum (Figure 1c). In case of pretreatment incubation time, no compelling improvement in delignification and saccharification yield between 4 and 6 h of incubation was observed (Figure 1d). Considering the economic perspective, 4 h incubation time was selected. Under optimized conditions, 3% NaOH, 5% WH biomass loading, at 100 °C for 4 h incubation resulted in the maximum yield of reducing sugar (523.6 mg/g of WH) after enzymatic hydrolysis. 

### 3.3. Ambient Conditions for Enzymatic Hydrolysis of NaOH Pretreated WH Biomass

**Figure 2:**
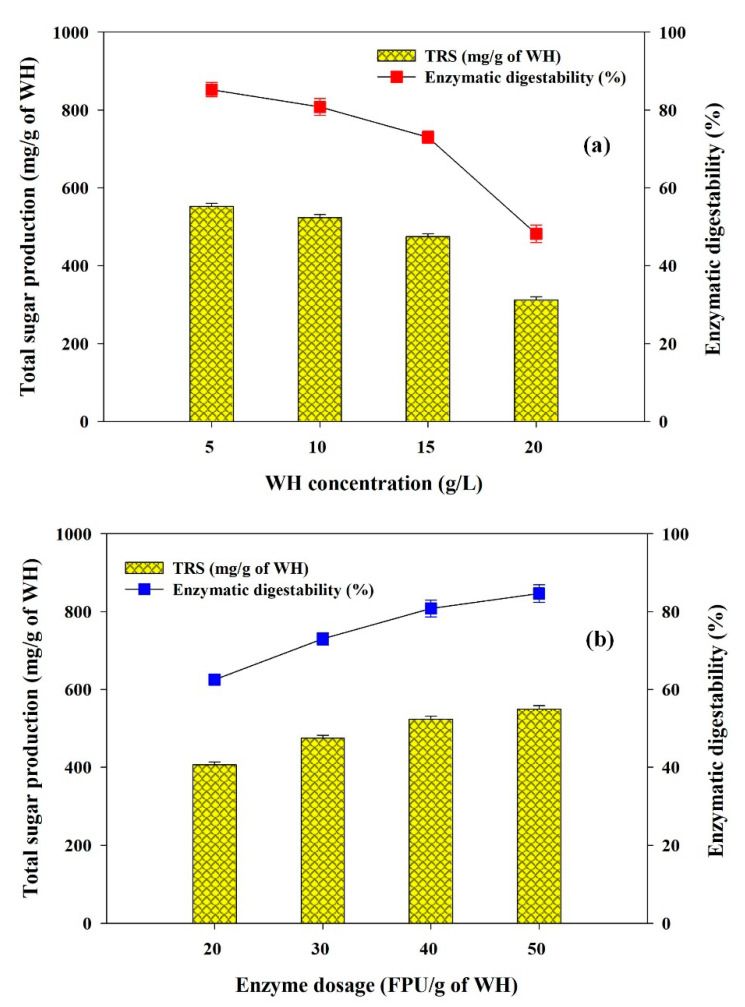
Effects of (**a**) substrate concentration (WH biomass) and (**b**) enzyme loading (FPU/g of WH) on enzymatic digestability and total reducing sugar production from WH.

Significant enzymatic saccharification of pretreated biomass is an important aspect to develop lignocellulosic biorefineries. It was observed that harsh chemical pretreatment produces inhibitors which severely affect enzymatic hydrolysis performance. Optimization of enzymatic hydrolysis parameters such as pH, temperature, substrate concentration and enzyme loading are essential to avoid excess usage of cellulase, and to get higher saccharification yields. It is also obligatory to make the biomass to biopolymers process more cost-effective and practically applicable. Significant hydrolysis of NaOH pretreated biomass was noted at 50 °C temperature and initial pH 5.0 of the buffer (data not shown) and thus maintained as optimal conditions for additional experiments. Further, the effect of WH loading from 5 g/L to 20 g/L and keeping the concentration of enzyme at 20 to 50 FPU/g of dry WH on total reducing sugar (TRS) production and hydrolysis yield was investigated. The optimal conditions to achieve better saccharification were 10 g/L of WH and 40 FPU/g of WH and released TRS about 523.6 mg/g of WH (Figure 2a,b). The obtained TRS yield was found to be significantly higher than other studies, including CaO_2_, combined microwave heating with 1% NaOH, and peracetic acid pretreated WH biomass which produces 325 mg/g, 306 mg/g and 430 mg/g of WH biomass, respectively, after enzymatic hydrolysis [27,28,29].

Moreover, the obtained saccharification yield was also found to be higher than the combined acid pretreatment and enzymatic hydrolysis under appropriate conditions which produce about 402.93 mg of reducing sugar [30].

### 3.4. Physicochemical Changes of Water Hyacinth after Pretreatment

**Figure 3:**
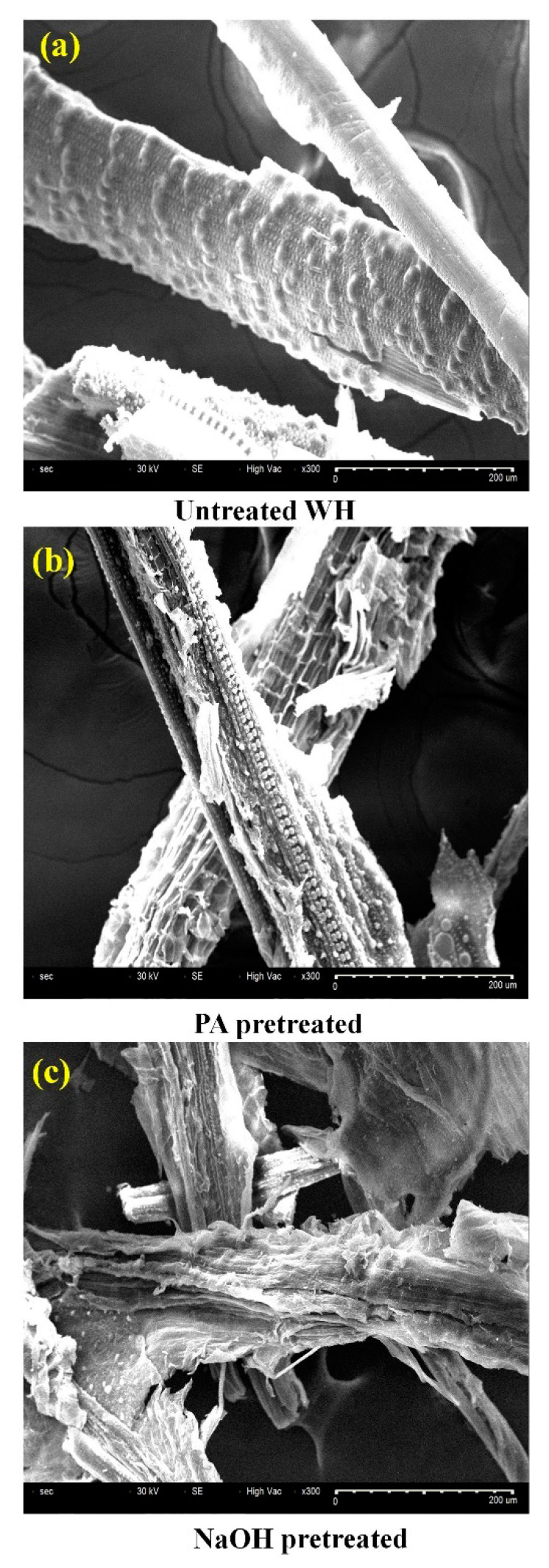
Scanning electron microscopy (SEM) micrographs of (**a**) untreated WH, (**b**) peracetic acid (PA) pretreated and (**c**) NaOH pretreated WH biomass under optimized conditions.

To get the molecular prospects of saccharification after chemical pretreatment, WH biomass was examined by using SEM, FTIR and XRD standard analytical tools. Initially, the surface morphological modifications were studied by taking SEM images before and after pretreatment. The untreated WH biomass showed a highly rigid compact structure, while PA and NaOH pretreated biomass showed rough scaly dispersed and distorted structures (Figure 3). NaOH pretreatment exhibited a higher destruction of surface morphology and increased porosity and surface area of biomass samples relative to PA pretreatment (Figure 3b,c).

This may be because of significant delignification and hemicellulose solubilization of biomass enhancing enzymatic accessibility.

**Figure 4:**
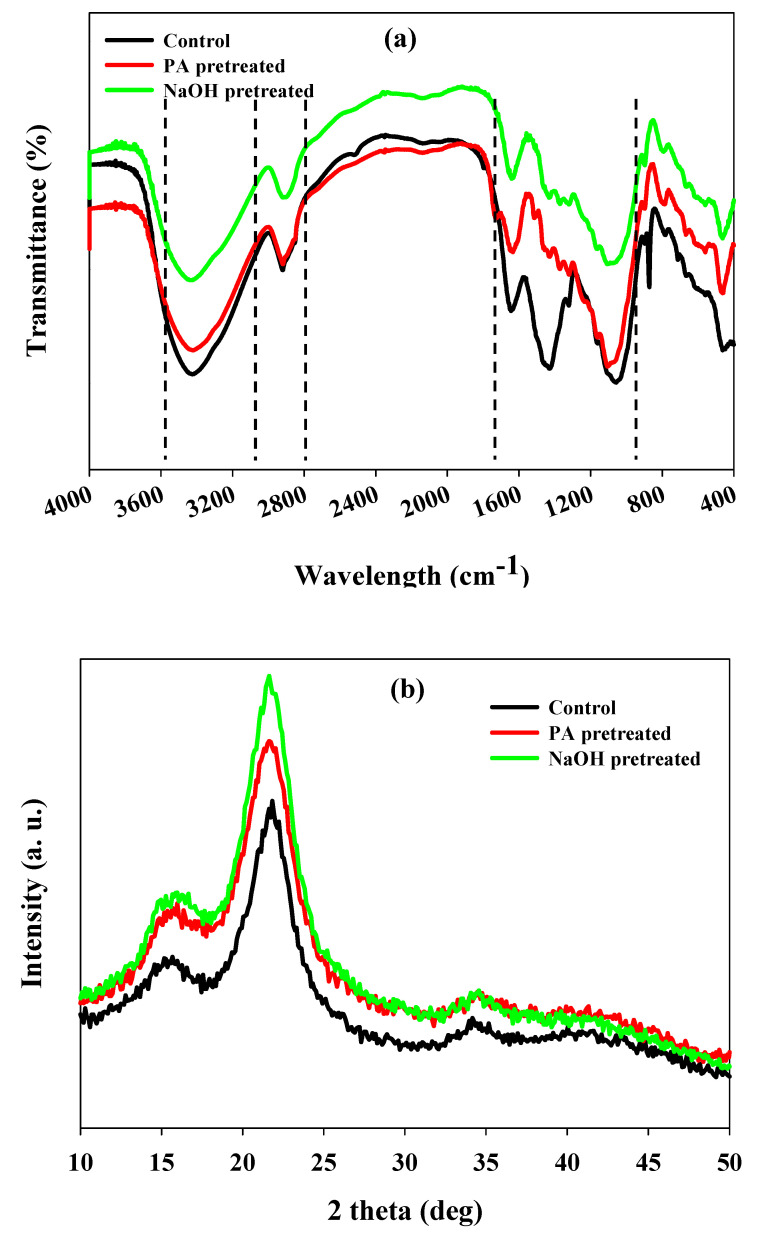
(**a**) Fourier transform infrared spectroscopic (FTIR) spectrum, (**b**) X-ray diffraction (XRD) pattern of untreated WH, peracetic acid pretreated and NaOH pretreated WH biomass under optimized conditions.

FTIR analysis was performed to analyze the chemical transformation of cellulose, lignin and hemicellulose of WH biomass before and after PA and NaOH pretreatment. The FTIR spectrum showed significant differences in the shape and transmittance of absorption band intensity. The stretched alteration in absorption band concentration at 2920–3330 cm^−1^ after chemical pretreatment reveals the dissociation of –OH stretch of lignin and –CH stretch, indicating cellulose disruption of WH biomass [31] (Figure 4a).

The maximum modification of peak at around 2930 cm^−1^ corresponds to C–H stretch of alkanes of structure of lignin was observed in NaOH pretreatment relative to PA and untreated WH biomass (Figure 4a). The results exhibited the significant removal of lignin in WH after NaOH pretreatment. Whereas, severe modification in band at 2914 cm^−1^ related to –CH_2_ stretching confers the dissociation of methyl or methylene groups of cellulose [17]. In addition, the reduced peak intensity at 1645 cm^−1^ portrays the confirmation of partial exclusion of hemicellulose [32]. A significant drop in peak intensities was observed at 1424, 1062, and 882 cm^−1^, concluding the lignin depolymerization and partial removal of hemicelluloses and lignin (Figure 4a) [33,34]. Overall, FTIR results suggest that NaOH pretreatment leads to structural transformation, with broadening of the peak indicating dissociation of intra and intermolecular hydrogen bonds, ultimately reducing the crystallinity of biomass.

X-ray diffractograms of untreated and chemically pretreated WH biomass are depicted in Figure 4b. This analysis gives clear evidence of pretreatment on crystallinity and the amorphous nature of cellulose of biomass by calculating the crystallinity index (CrI). NaOH (58.8%) and PA (52.8%) pretreatment showed significant increases in CrI as compared to untreated (41.4%) WH biomass (Figure 4b). Significant enhancement in CrI of NaOH pretreated biomass revealed higher delignification, partial removal of hemicelluloses and an increase in the cellulose content of WH biomass, which is more accessible. A similar line of observations where increase in CrI of WH biomass after chemical pretreatment were recorded by some investigators [31,35].

### 3.5. PHB Production Using WH Hydrolysates

This research work is aiming to utilize water hyacinth as a potential biomass resource for PHB synthesis using *R. eutropha* as the fermenting organism. To keep away from competition with food production by utilizing abundant, sustainable, renewable WH biomass resources makes the process more ecofriendly and cost-effective [9]. The resulting enzymatic hydrolysates after PA and NaOH pretreatment by keeping TRS concentration (20 g/L) was applied for biopolymer production. The results showed less sugar consumption, bacterial growth and less PHB accumulation in PA as compared to NaOH pretreated WH hydrolysates. This may be because of the presence of more 5C sugars and fermentation inhibitors in PA pretreated hydrolysates. The higher assimilation of sugars of about 70% and significant bacterial growth in DCW (8.40 g/L), PHB accumulation (67.5%) and significant PHB titer (5.67 g/L) was observed in NaOH pretreated WH hydrolysates (Table 3). In addition, the kinetics parameters of bacterial growth and PHB production were compared by taking SH hydrolysates, which have a similar sugar composition with NaOH pretreated WH hydrolysates. The results showed that bacterial growth and PHB production were quite similar (Table 3); however, results also suggest that 5C sugars were not assimilated effectively in both cases by *R. eutropha*.

#### Effects of Supplementation of Cheap Nitrogen Source on PHB Production

**Figure 5:**
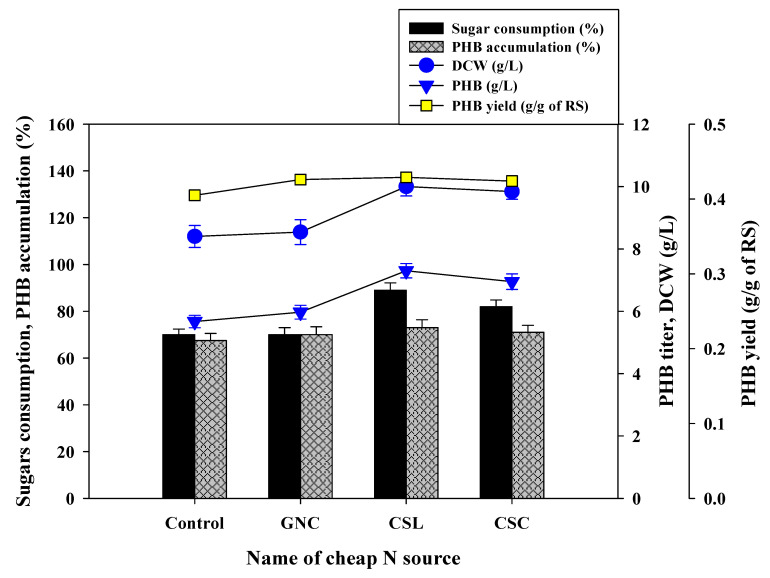
Effects of supplementation of cheap nitrogen sources with NaOH pretreated WH enzymatic hydrolysates on bacterial cell growth and PHB production.

Microbial PHB production research is still at an initial stage to make the process more cost-effective, with the optimization of parameters such as selection of microbial strains, synthesis of PHB metabolic pathways, C and N source and inexpensive PHB extraction procedures [2]. In this study, to economize and enhance the assimilation of sugars in WH enzymatic hydrolysates and PHB production, further study was concentrated on supplementation of cheap N sources and checking their performance. The results showed that GNC was not effective to enhance sugar assimilation; however, a very slight increase in DCW and PHB production was noticed (Figure 5). CSC showed a moderate increase in sugar assimilation and PHB production while a higher performance in both parameters was observed in CSL. With CSL, *R. eutropha* exhibited a higher sugar assimilation (89%) with enhanced bacterial cell growth (DCW 10.0 g/L), and effective PHB titer (7.3 g/L) and yield of PHB at around 0.429 g/g of sugar (Figure 5). There is a substantial increase in PHB titer at about 25% and 21% DCW was recorded as compared to only NaOH pretreated enzymatic WH hydrolysates.

The complex mixture of nutrients in CSL and CSC enhanced bacterial cell growth and favored PHB accumulation. Further detailed investigation is essential to know the exact mechanism. However, supplementation of cheap N source with WH hydrolysates can make the process more cost effective, and practically applicable efficient PHB production at large scale levels. Recently, Dalasso et al. [36] used a combination of sugarcane vinasse and molasses as a carbon source for PHB production. The maximum PHB accumulation of 56% and PHB titer of 11.70 g/L was observed. They have stated that, during PHB production progression, N content in sugarcane molasses is accountable for higher cell growth and favorable for PHB accumulation. Moreover, the obtained PHB accumulation and yield was found higher than other studies utilizing water hyacinth biomass by using *R. eutropha* MTCC 1472 [10]; by *Ralstonia eutropha* MTCC 8320 [9]; using bacterial isolates [37] and using *Bacillus cereus* and *Bacillus subtilis* [38]. The obtained results of PHB production by *R. eutropha* were compared and found satisfactory with the literature (Table 4). It was hypothesized that, during pretreatments, less utilization of sugars by *R. eutropha* was observed which could be because of the existence of 5C sugars or fermentation inhibitors generated in the hydrolysates. Moreover, in chemically treated WH hydrolysates, detoxification is essential for the removal of fermentation inhibitors and to enhance the assimilation of sugars and their conversion into PHB.

### 3.6. PHB Characterization

**Figure 6:**
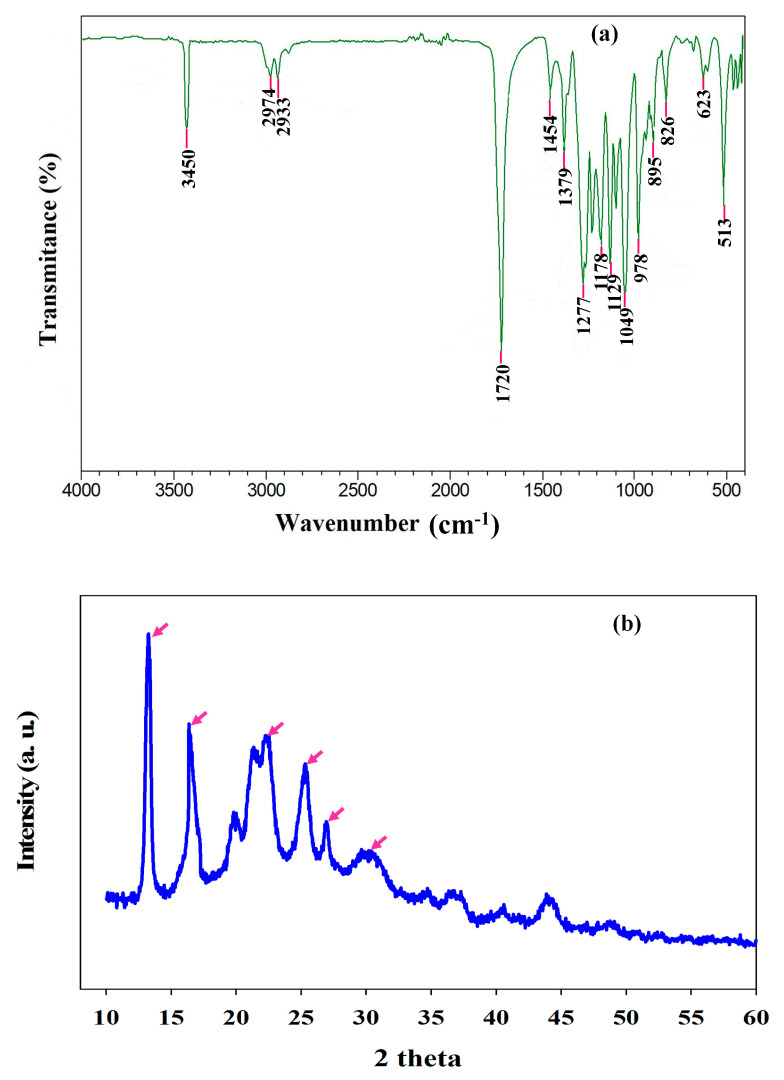
(**a**) FTIR analysis and (**b**) XRD pattern, of the PHB produced by *Ralstonia eutropha* using alkaline pretreated WH hydrolysates.

FTIR analysis was applied to reveal the chemical functional groups of the extracted polymer. The bands at 1722 cm^−1^ and 1278 cm^−1^ in produced PHB correspond to C=O and C–O stretching bands of standard PHB (Figure 6a) [17]. The peak in the region of 2900 cm^−1^ ascribed to C–H stretching vibration of alkanes, which is a component of the PHB (Figure 6a) [43]. The band at 1454 cm^−1^ assigned to the asymmetrical deformation of C–H bond in –CH_2_ together with –CH_3_ groups at 1380 cm^−1^ [9]. The peak at 1000–1300 cm^−1^ related to C–O stretching of the ester group; similarly, the absorption peak at 978 to 513 cm^−1^ represents the existence of the ester bonds present in PHB configurations (Figure 6a). The foregoing results suggest that the spectrum of the extracted PHB is consistent with that of standard PHB and literature.

The crystalline structure of the extracted biopolymer was studied by XRD examination. The XRD spectrum showed the distinctive peak values of 2θ = 13.2, 16.4, 22.2, 25.3 and small intensity peaks at 26.8 and 30.3° (Figure 6b). The obtained higher and intense peaks exhibiting the extracted PHB have higher degrees of crystallinity. The obtained results of XRD analysis of the produced PHB were closely matched to PHB produced by *R. eutropha* [9,17].

### 3.7. Thermal (TGA and DSC) Analysis of Produced PHB

**Figure 7:**
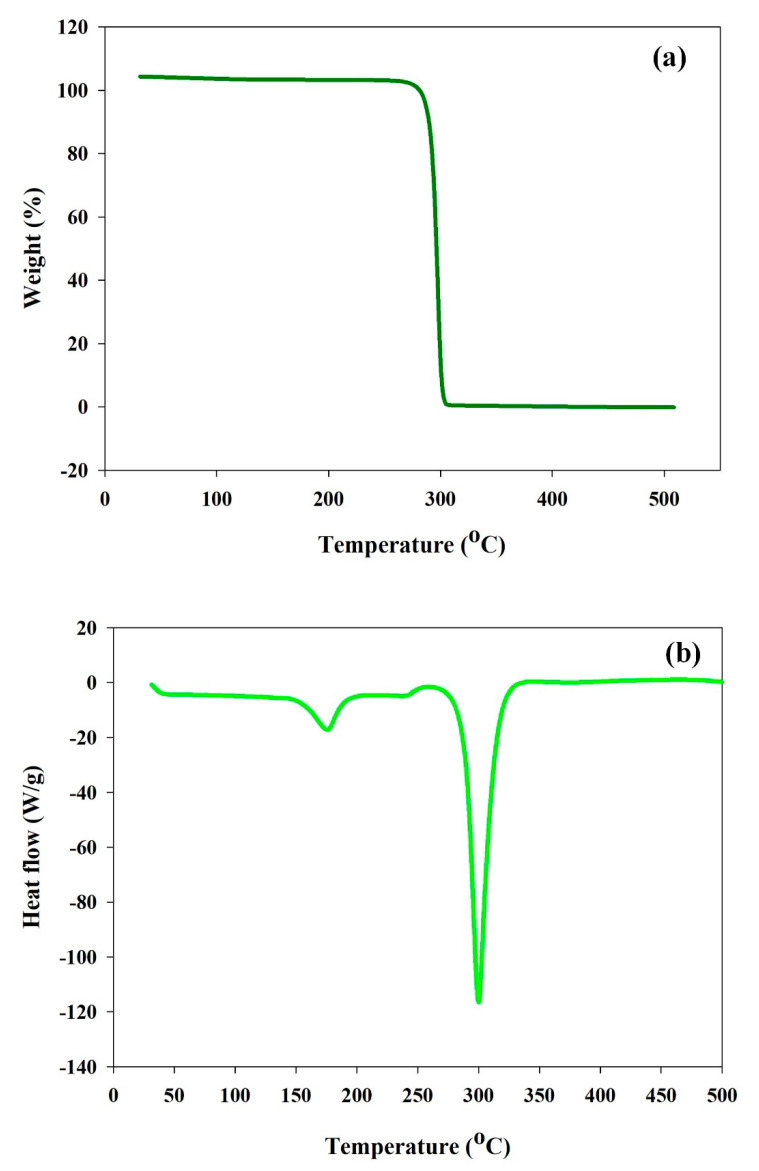
(**a**) Thermo gravimetric analysis (TGA) and (**b**) differential scanning calorimetry (DSC) analysis of the PHB produced by *Ralstonia eutropha* using alkaline pretreated WH hydrolysates.

Determination of thermal stability and melting temperature are vital properties of biopolymers. Thermo gravimetric analysis (TGA) studies showed elevated decomposition temperature (*T_d_*) at 292 °C (Figure 7a) and no ashes were observed during thermal degradation of the extracted PHB. Complete degradation of carbonaceous residues disappeared at 338 °C (Figure 7a). Similar results have been recorded in PHB synthesized by *Bacillus sphaericus* NII 0838 [44]. Differential scanning calorimetry was employed to measure the melting temperature (*T_m_*) and glass transition temperature (*T_g_*). The analysis exhibited that the extracted PHB polymer contains the *T_m_* of 175 °C, and *T_g_* of 12 °C which is quite similar with *T_g_* (9–15 °C) and *T_m_* (175 °C) of the standard PHB (Figure 7b). PHB produced from *Burkholderia cepacia* using detoxified wood hydrolysate showed similar (*T_m_*) of and (*T_g_*) values of 174.4 °C, and 7.31 °C, respectively, with a lower decomposition temperature (*T_d_*) of 268.6 °C [45]. The obtained result revealed that the synthesized PHB is a thermally stable and crystalline homopolymer [9,10].

## 4. Conclusions

The present study exploits the conversion of aquatic weed water hyacinth biomass hydrolysates into Poly-β-hydroxybutyrate (PHB) by *Ralstonia eutropha*. Alkaline pretreatment and their conditions were optimized to enhance the saccharification of WH biomass with the aim to make the process more economical and environmentally-friendly in the real field. Further, optimization of growth parameters and supplementation of cheap N sources to improve PHB production was studied. The outcome of this study reveals the increased PHB titer of 7.30 g/L and PHB yield of 0.429 g/g of reducing sugar. The bacteria-produced PHB was analyzed by different standard analytical techniques, and the obtained results are consistent with that of standard PHB. Additionally, to economize, supplementation of CSL was found to be the best strategy to enhance PHB production. Further studies will be devoted towards the optimization of conditions and bioreactor studies to make large-scale production using WH biomass. Results obtained from this study showed the feasibility of the noxious and inexpensive weed WH biomass for sustainable bioplastic production.

## Figures and Tables

**Table 1:** Polyhydroxybutyrate (PHB) production media composition using water hyacinth (WH) hydrolysates and synthetic hydrolysates [17].

Name of Component	Concentration (g/L)
NaH_2_PO_4_	3.60
Na_2_HPO_4_	2.84
K_2_SO_4_	3.49
NaOH	0.40
Yeast extract	0.20
MgSO_4_·7H_2_O	0.39
CaCl_2_	0.06
(NH_4_)_2_SO_4_	0.10
CuSO_4_·5H_2_O	0.005
ZnSO_4_·7H_2_O	0.024
MnSO_4_·H_2_O	0.024
FeSO_4_·7H_2_O	0.15
WH hydrolysates ^#^	20.0
SH hydrolysates ^#^	20.0

^#^ The hydrolysate composition of mainly glucose (g/L), xylose (g/L), and arabinose (g/L).

**Table 2:** Effects of alkaline and peracetic acid pretreatment on WH biochemical constituents, delignification, and hydrolysis yield and glucose yield after enzymatic hydrolysis.

Type of Pretreatment	Pretreatment Conditions	WH Biochemicals Constituents (%)			Delignification (%)	TRS (mg/g of WH)	Hydrolysis Yield (%)	Glucose Yield (%)
		Cellulose	Hemi-cellulose	Lignin				
Control	No pretreatment	29.15 ± 1.25	32.66 ± 1.28	10.25 ± 0.68	ND	65.80 ± 1.50	10.65 ± 0.51	13.7 ± 0.25
NaOH	2% NaOH at 100 °C for 3 h	42.25 ± 1.85	22.86 ± 0.89	5.11 ± 0.32	50.2 ± 1.25	418.0 ± 3.87	64.32 ± 0.68	75.0 ± 0.75
Peracetic acid (PA)	2% Peracetic acid at 100 °C for 3 h	37.86 ± 1.95	24.50 ± 1.15	6.15 ± 0.30	40.3 ± 1.29	312.7 ± 3.56	50.12 ± 0.62	60.5 ± 0.65

Values are the mean of three experiments; (±) standard error (SE) by one-way ANOVA with Tukey–Kramer Multiple Comparisons Test.

**Table 3:** Sugar consumption, *R. eutropha* growth and PHB production kinetics parameters using chemically pretreated WH enzymatic hydrolysates and synthetic hydrolysate (SH) (concentration: 20 g/L).

Parameters	NaOH	PA	SH
Fermentation time (h)	36	36	36
TRS (initial) (g/L)	20 ± 0.55	20 ± 0.65	20 ± 0.66
TRS (after) (g/L)	6.0 ± 0.15	8.4 ± 0.14	4.2 ± 0.12
Total Sugar consumption (%)	70.4 ± 1.25	60.5 ± 0.98	80.0 ± 1.00
DCW (g/L)	8.40 ± 0.35	7.25 ± 0.35	9.72 ± 0.48
PHB/DCW content (%)	67.5 ± 1.88	62.5 ± 1.65	70.0 ± 1.45
PHB (g/L)	5.67 ± 0.25	4.53 ± 0.25	6.80 ± 0.22
Q_p_ gPHB/L/h	0.157 ± 0.001	0.125 ± 0.001	0.188 ± 0.002
PHB yield (g/g)	0.405 ± 0.001	0.377 ± 0.001	0.425 ± 0.002

Values are the mean of three experiments: (±) standard error (SE) by one-way ANOVA with Tukey–Kramer Multiple Comparisons Test.

**Table 4:** Comparison of PHB accumulation and PHB production by *Ralstonia eutropha* strains using different biomass resources as a substrate.

Name of Substrate	Microorganism	Operation Mode	PHB Content (%)	PHB Concentration (g/L)	Reference
Water hyacinth	*R. eutropha* ATCC 17699	Batch	73	7.30	This study
Water hyacinth	*R. eutropha* MTCC 1472	Batch	58.0	7.0	[10]
*Partheniumhysterophorus* Pentose-rich hydrolysate Hexose-rich hydrolysate	*R. eutropha* MTCC 8320	Batch	8.03 17.93	0.24 0.60	[9]
*Eicchornia crassipes* Pentose-rich hydrolysate Hexose-rich hydrolysate	*R. eutropha* MTCC 8320	Batch	8.11 21.62	0.30 0.96	[9]
Waste office paper	*R. eutropha* NCIMB 11599	Batch	57.5	3.93	[39]
*Sargassum* sp. seaweed hydrolysate	*C. necator* PTCC 1615	Batch	74.4	3.93	[40]
Wheat waste biomass	*R. eutropha* ATCC 17699	Batch	74.0	7.85	[8]
Paddy straw	*R. eutropha* MTCC 1472	Batch	37.55	5.19	[41]
Rice paddy straw	*R. eutropha* ATCC 17699	Batch	75.45	11.42	[17]
Bagasse hydrolysate	*R. eutropha* ATCC 17699	Batch	65	3.9	[42]
